# Bilateral Field Advantage in Visual Enumeration

**DOI:** 10.1371/journal.pone.0017743

**Published:** 2011-03-23

**Authors:** Jean-François Delvenne, Julie Castronovo, Nele Demeyere, Glyn W. Humphreys

**Affiliations:** 1 Institute of Psychological Sciences, University of Leeds, Leeds, United Kingdom; 2 University of Louvain, Louvain-la-Neuve, Belgium; 3 School of Psychology, University of Birmingham, Birmingham, United Kingdom; University of Minnesota, United States of America

## Abstract

A number of recent studies have demonstrated superior visual processing when the information is distributed across the left and right visual fields than if the information is presented in a single hemifield (the *bilateral field advantage*). This effect is thought to reflect independent attentional resources in the two hemifields and the capacity of the neural responses to the left and right hemifields to process visual information in parallel. Here, we examined whether a bilateral field advantage can also be observed in a high-level visual task that requires the information from both hemifields to be combined. To this end, we used a visual enumeration task—a task that requires the assimilation of separate visual items into a single quantity—where the to-be-enumerated items were either presented in one hemifield or distributed between the two visual fields. We found that enumerating large number (>4 items), but not small number (<4 items), exhibited the bilateral field advantage: enumeration was more accurate when the visual items were split between the left and right hemifields than when they were all presented within the same hemifield. Control experiments further showed that this effect could not be attributed to a horizontal alignment advantage of the items in the visual field, or to a retinal stimulation difference between the unilateral and bilateral displays. These results suggest that a bilateral field advantage can arise when the visual task involves inter-hemispheric integration. This is in line with previous research and theory indicating that, when the visual task is attentionally demanding, parallel processing by the neural responses to the left and right hemifields can expand the capacity of visual information processing.

## Introduction

Enumerating visual objects from a visual scene is a task the human brain must continuously perform. When individuals are asked to determine the numbers of briefly presented visual items, differences in the slope accuracy are typically found between small versus large numbers [1], [2]. Enumeration remains fairly accurate up to four items but deteriorates rather dramatically with larger numbers. The process of enumerating small numbers is known as *subitizing* [3], while enumerating large numbers is thought to either reflect *counting* (when sufficient presentation time is given) or to engage the *approximate number system* (when the items are briefly presented) [4].

In a typical visual enumeration task, the items are randomly presented on a computer screen, with some items inevitably falling in the left visual field and others in the right visual field. Given that the information from the left visual field generates initially a greater neural response in the right visual cortex and the information from the right visual field in the left visual cortex, the brain needs to integrate all that information across the hemispheres to represent a single quantity. The corpus callosum allows processing occurring in one hemisphere to be transmitted to and integrated with processing occurring in the other hemisphere [5]. The effect of inter-hemispheric integration on visual enumeration, however, is currently unknown and two predictions can be made.

On the one hand, recent findings have suggested temporal and qualitative differences between the integration of visual information within and across the hemifields, with within-hemifield integration preceding [6], [7] and being more efficient [8], [9] than across-hemifield integration. For instance, Large and colleagues [6] found that the same regions in the lateral occipital cortex (LO) respond both to the upper and lower visual fields, but with a clear contralateral preference. Using the technique of fMRI adaptation, the authors found a greater adaptation to vertical translations of faces within the same hemifield than across-hemifield translations, suggesting that the upper and lower visual representations are combined in the contralateral LO prior to the integration of the left and right representations. Consistent with this, the completion of illusory contours [9] and processes of perceptual grouping [8] are stronger when the stimuli appear within the same hemifield than when they cross hemifields. Accordingly, as across-hemifield integration is required in an enumeration task when the items are split between the two hemifields, we may predict a *unilateral field advantage* in visual enumeration, namely better performance when the items are unilaterally presented as when they are bilaterally displayed.

On the other hand, another line of research has suggested that there exist independent attentional resources for the left and right hemifields [10], [11] and that parallel processing by the neural responses to the left and right hemifields can expand the capacity of visual information processing. This has been reported in a number of attentional demanding visual tasks, such as object tacking [12], short-term memory for spatial locations [13], item identification [14] and orientation discrimination and detection [15] among other visual tasks. These tasks are better performed when the items are distributed across the left and right visual fields as when they were all displayed within a single hemifield. Contrary to visual enumeration, the tasks employed in those studies do not require the information from the left and right visual fields to be combined. Rather, visual representations from both fields could be processed independently by each of the hemispheres and still support task performance. However, if such a bilateral advantage is a general feature of selective attention, as previous findings seem to suggest [12]–[15], we may predict a *bilateral field advantage* in visual enumeration only when the task requires a certain amount of attentional resources. More precisely, a bilateral field advantage may be observed beyond the subitizing range, that is to say when at least four items have to be enumerated. In the present study, the effect of distributing visual items across the two hemifields on visual enumeration was directly tested by pitting the unilateral and bilateral field advantage hypotheses against each other.

## Experiment 1

In this experiment two to eight dots were quickly presented on a computer screen and fell either all in the same visual field (unilateral condition) or in the two visual fields (bilateral condition). Participants were asked to keep their eyes on the centre of the screen and to enumerate the dots as accurately as possible. The crucial question was whether visual enumeration would benefit, or alternatively suffer from the bilateral presentation.

### Method

#### Participants

A total of 20 volunteers (13 women), aged between 19 and 37 years (mean = 24) took part in the experiment. In all experiments, the participants had self-reported normal or corrected-to-normal vision, and they were naïve to the experimental aims. They provided written and informed consent before experiments, and all procedures were approved by the ethic committee of the University of Leeds. They were offered £6 in exchange for their time.

#### Stimuli and procedure

**Figure 1:**
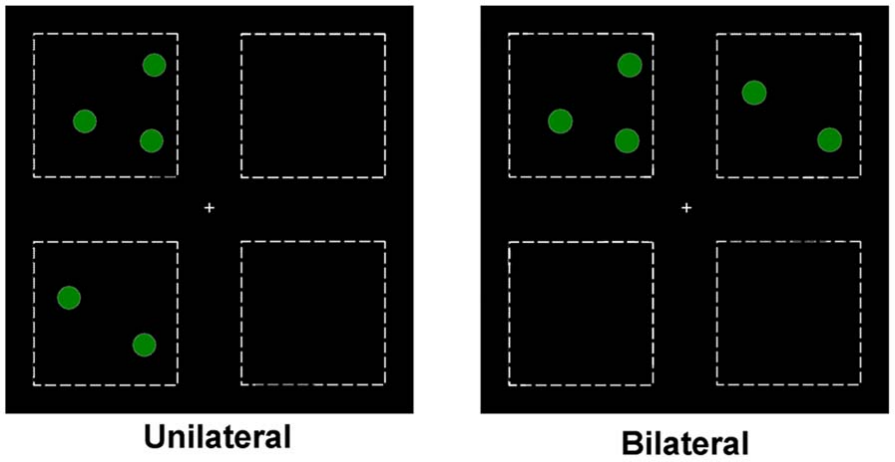
Samples of displays used in Experiment 1. In the unilateral condition, the items appeared in two quadrants from the same hemifield (either the left or right hemifield). In the bilateral condition, the items appeared in two horizontally symmetrical quadrants from different hemifields. Note that dotted lines that delimitate the four quadrants are shown for illustration purpose only. The quadrants were invisible in the experiment.

The stimuli were presented on the 17-in. monitor of a Pentium-based computer running E-Prime 1.1 software (Psychology Software Tools, Inc. www.pstnet.com/eprime). Participants viewed the computer screen at eye level from a distance of approximately 60 cm. Eye movements were not monitored, but participants were encouraged to keep their eyes focused on the centre of the screen throughout the experiment. The resolution of the screen used was 1024×768 pixels and the screen background was black. The displays consisted of four invisible quadrants (subtending 4.43°×4.43° each) placed around a central fixation point and separated vertically and horizontally by 2.32°. Green dots (0, 130, and 0 on red, green, and blue phosphors respectively) with a diameter of 10 pixels (0.34°) were used as stimuli. The dots were placed randomly within two of the four quadrants with a minimum centre-to-centre spacing between dots of 38 pixels (1.3°). The number of dots within one quadrant ranged from 1 to 4. The total number of dots on the screen varied therefore between 2 and 8, with all possible combinations evenly used. There were two conditions: unilateral and bilateral (see Figure 1). In the unilateral condition, the dots appeared in two quadrants from the same visual field (upper-left/lower-left or upper-right/lower-right). In the bilateral condition, they appeared in two horizontally symmetrical quadrants from different hemifields (upper-left/upper-right or lower-left/lower-right).

The experiment was conducted in a quiet and dimly illuminated room. A single trial started with a blank screen for 1000 ms, followed by a central fixation point (a small white cross subtending 0.61°×0.61°) for 500 ms. The stimulus display was then presented for 150 ms, followed by a blank screen that endured until a response was made. Participants responded by pressing the space bar and simultaneously speaking their response (for a similar procedure, see [16]–[18]). Participants were then prompted to encode their response by pressing a number key on the computer key pad. Twenty practice trials were completed followed by 8 blocks of 56 test trials (2 conditions×7 numbers of dots×32 trials). All conditions were randomized within blocks. After each block, participants were given the opportunity to take a break during which they were shown their correct response rate and mean response latency. They were politely warned if their accuracy was lower than 60%. Participants then pressed the space key to continue.

### Results and Discussion

**Figure 2:**
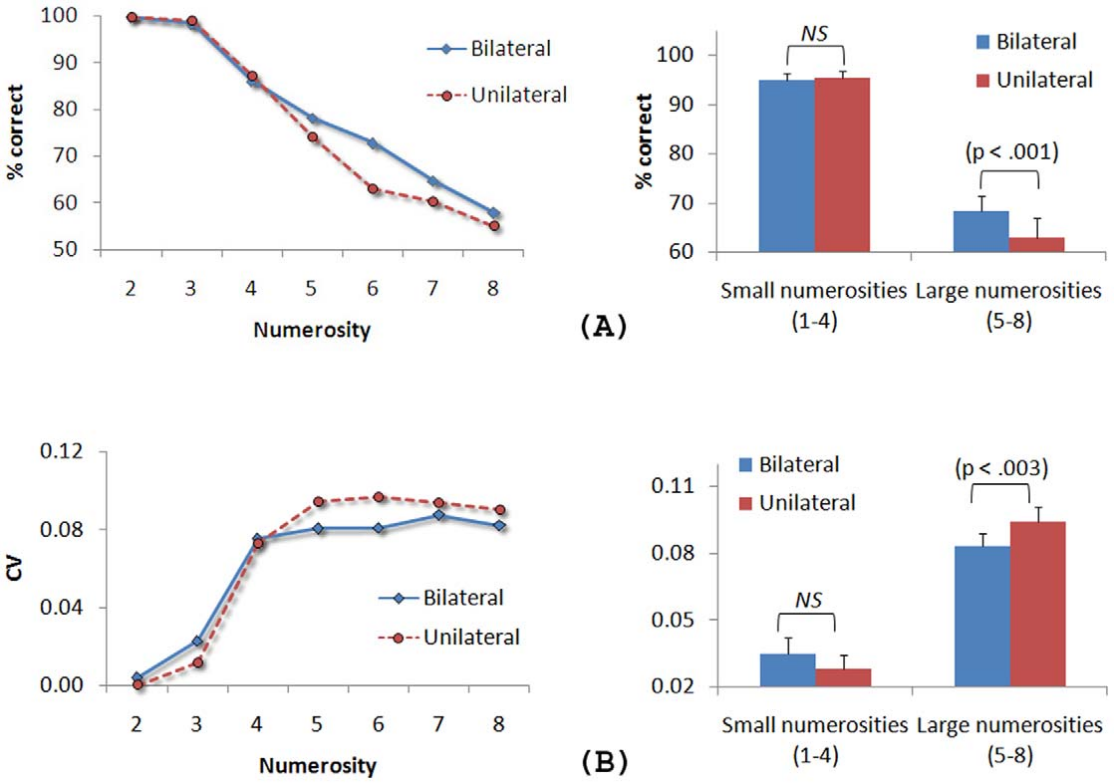
The results of Experiment 1. (A) Percentage of correct responses and coefficients of variation as a function of condition and numerosity. (B) Averaged percentage of correct responses and coefficients of variation divided across small and large numerosities. The p value is shown when the difference between the bilateral and unilateral conditions is significant. ‘NS’ (i.e., ‘non significant’) is shown when the difference is not significant. The error bars represent ±1 standard error of the mean. No error bars are shown in (A) to preserve the readability of the data.

To avoid eye movements, the stimulus displays were presented only for a very short time (150 ms). Therefore, we used accuracy (percentage of correct responses) as a measure of performance as well as the coefficient of variation (CV) (the ratio of the standard deviation and the mean response) as a measure of response precision. In all analyses, Greenhouse-Geisser corrections for non-sphericity were applied where appropriate. The results are plotted in Figure 2.

A 2 (condition: unilateral, bilateral)×7 (numerosity: two – eight) ANOVA (repeated measures) on accuracy revealed a main effect of condition, F(1, 19) = 7.92, MSE = 0.007, p<.01, with better performance in the bilateral condition than in the unilateral condition, a main effect of numerosity, F(2.98, 56.22) = 43.60, MSE = 0.056, p<.001, where more errors were made as the number of dots increased, and a significant condition × numerosity interaction, F(302, 57.34) = 2.74, MSE = 0.011, p<.05. The interaction revealed a condition effect only for the larger numerosities (i.e., five, six, seven and eight) (p<.001) (see Figure 2a).

Possible left-right visual field asymmetries were also examined. Accuracy in the left visual field was significantly higher than in the right visual field, t(19) = 2.36, MSE = 0.013, p<.05. Previous investigations on visual field asymmetries for enumeration processes have provided controversial results. Some studies have revealed a left visual field advantage for enumeration [19]–[21], whereas other neuroimaging [22], [23] and neuropsychological [24] studies have found equivalent enumeration performance in both hemifields. More research is needed to clarify this issue and this will not be further discussed.

Finally, we asked whether the variability in responses also varied between the unilateral and bilateral conditions. Past research has shown that when more than 3 or 4 items are briefly presented, the approximate number system is engaged and both the mean and the standard deviation of responses increase linearly and in direct proportion as a function of numerosity, resulting in constant coefficients of variation (CVs) [25]–[27]. A 2 (condition) ×7 (numerosity) ANOVA (repeated measures) on CVs revealed no effect of condition (p>.24), a main effect of numerosity, F(3.17, 60.24) = 46.25, MSE = 0.002, p<.001, and a significant condition × numerosity interaction, F(6, 114) = 2.20, MSE = 0.000, p<.05. Figure 2b shows constant CVs of about 0.08 for numerosities greater than four in both conditions (p>.8), yielding evidence that the approximate number system was engaged in the task [25]–[27]. Furthermore, whereas the mean CVs for small numerosities (2–4) did not differ between the bilateral and unilateral conditions (p>.15), the mean CVs for larger numerosities (5–8) were significantly smaller in the bilateral condition (CV = 0.083) than in the unilateral condition (CV = 0.094), F(1, 19) = 11.92, MSE = 0.000, p<.003. This indicates less variability, thus higher precision, in responses when the to-be-enumerated items were split across the left and right visual fields.

Those results show that visual enumeration is more accurate and more precise when the items are displayed in the two visual fields relative to when they appear within the same hemifield. This bilateral field advantage was observed when more than four objects had to be enumerated, suggesting that this bilateral effect occurs when sufficient attentional resources are requisite. This is consistent with the notion of independent resources in the left and right hemifields [10], [11] and with the findings that parallel processing by the neural responses to the left and right hemifields can expand the capacity of visual information processing [12]–[15]. Furthermore, the present work extends those findings by showing that when the information needs to be integrated across the two hemifields, the initial parallel processing still benefits the task.

## Experiment 2

**Figure 3:**
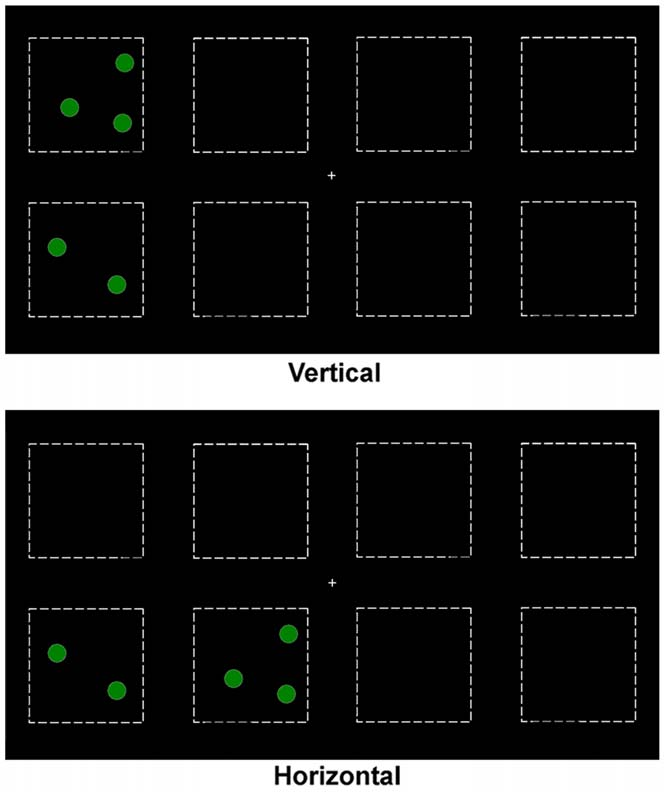
Samples of displays used in Experiment 2. In the vertical condition, the dots appeared in two vertically aligned quadrants within a single hemifield. In the horizontal condition, the dots appeared in two horizontally symmetrical quadrants within a single hemifield. Note that dotted lines that delimitate the quadrants are shown for illustration purpose only. The quadrants were invisible in the experiment.

In Experiment 1, the dots were vertically aligned in the unilateral condition, whereas they were horizontally aligned in the bilateral condition. Therefore, the possibility that the bilateral field advantage in visual enumeration actually reflects a horizontal advantage in number processing remains. To control for this, the same two spatial arrangements of dots (i.e., vertical versus horizontal) were used in Experiment 2, but were always presented within a single hemifield (see Figure 3, and [12] for a similar procedure). If the bilateral field advantage can be explained by the horizontal alignment of the dots, then we would expect better performance when the dots are horizontally aligned than when they are vertically aligned.

### Method

#### Participants

Seventeen new volunteers (11 women), aged between 21 and 39 years (mean = 26) took part in the experiment.

#### Stimuli and procedure

This experiment replicated Experiment 1 except that the four quadrants shifted 6.58° to the left or right of fixation so that all four quadrants fell within a single hemifield (Figure 3). In the *vertical condition*, the dots appeared in two vertically aligned quadrants either on the far left, near left, near right, or far right. In the *horizontal condition*, the dots appeared in two horizontally aligned quadrants either on the top left, bottom left, top right, or bottom right. Twenty practice trials were followed by 16 blocks of 56 trials (2 conditions ×2 hemifields ×7 numbers of dots ×32 trials).

### Results and Discussion

**Figure 4:**
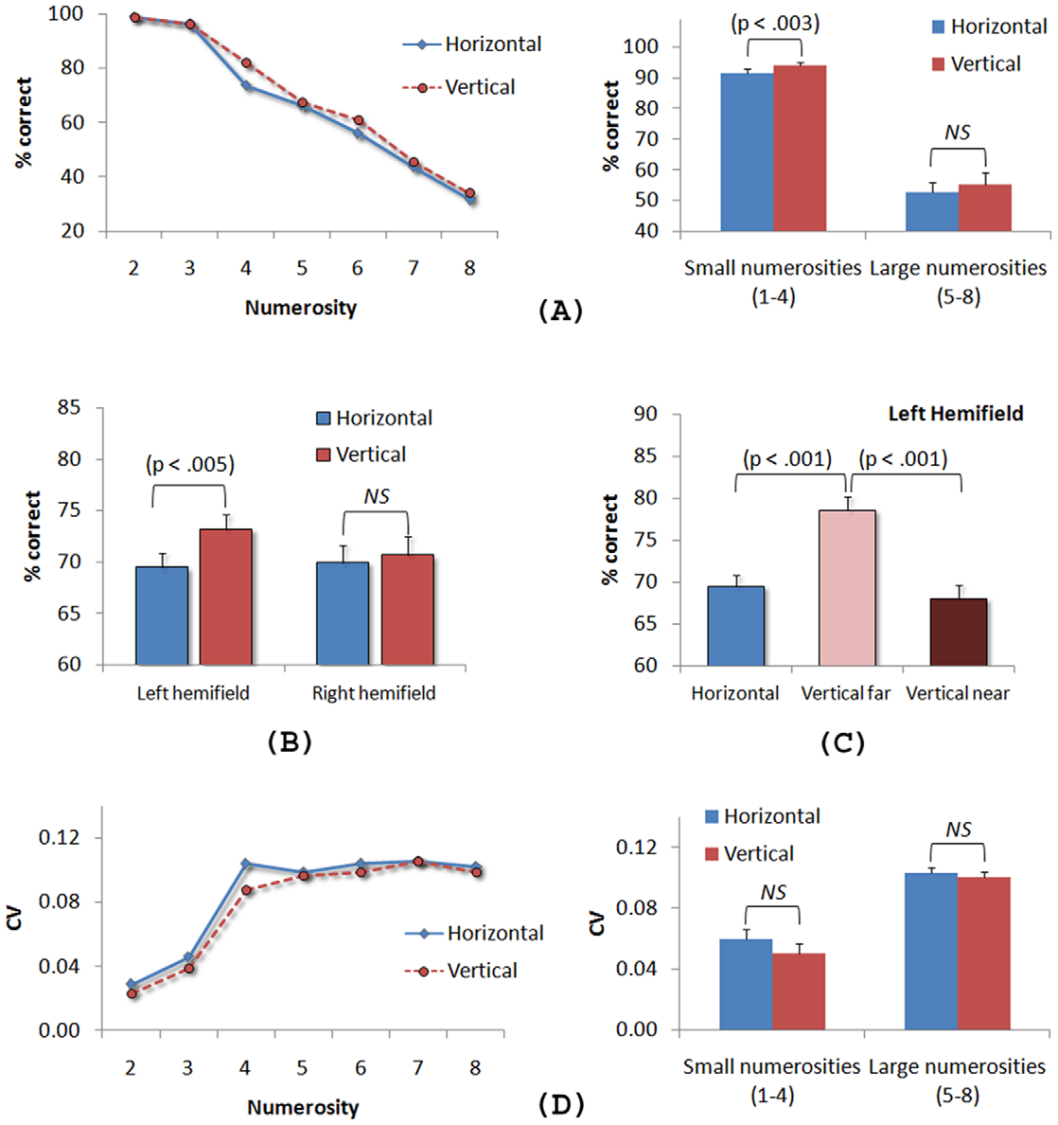
The results of Experiment 2. (A) Percentage of correct responses and coefficients of variation as a function of condition and numerosity. (B) Averaged percentage of correct responses and coefficients of variation divided across small and large numerosities. (C) Percentage of correct responses as a function of condition and hemifield. (D) Percentage of correct responses in the left horizontal, far left vertical and near left vertical alignments. The error bars represent ±1 standard error of the mean.

A 2 (condition: vertical, horizontal) ×7 (numerosity) ANOVA (repeated measures) on accuracy revealed a main effect of condition, F(1, 16) = 10.86, MSE = 0.003, p<.005, a main effect of number of dots, *F*(2.36, 38.24) = 109.18, MSE = 0.048, p<.001, but no interaction (p>.11). The condition effect was observed only for the small numerosities (2–4), *F*(1, 16) = 11.77, MSE = 0.001, p<.003 (see Figure 4a). Surprisingly, it was the vertical condition that yielded better performance (72% and 69% in the vertical and horizontal conditions, respectively). We conducted a 2 (condition) ×2 (hemifield) ANOVA (repeated measures) to see whether the vertical advantage was present in both hemifields. The results revealed a main effect of condition, F(1, 16) = 10.34, MSE = 0.001, p<.005, no effect of hemifield (p>.3), and a significant condition × hemifield interaction, *F*(1, 16) = 14.75, MSE = 0.000, p<.001, indicating a vertical advantage in the left hemifield only (see Figure 4b). To further explore this effect, we carried out separate analyses for each of the four vertical positions (i.e., far left, near left, near right, and far right) and the results showed a vertical advantage only when the dots were displayed in the far left quadrants, t(16) = −8.66, p<.001 (see Figure 4c). This finding suggests that the vertical advantage observed in this experiment may simply reflect a left hemifield bias in enumeration [19]–[21]. Finally, the 2 (condition) ×7 (numerosity) ANOVA (repeated measures) on CVs revealed no effect of condition (p>.05), a main effect of numerosity, F(3.20, 51.27) = 51.72, MSE = 0.001, p<.001, and no significant condition × numerosity interaction (p>.54). Figure 4d shows constant CVs of about 0.10 for numerosities greater than four in both conditions (p>.15). The mean CVs did not differ between the horizontal and vertical conditions for both small numerosities (p>.08) and large numerosities (p>.28). The critical finding in Experiment 2 was the absence of a horizontal advantage in visual enumeration. Thus, the bilateral field advantage observed in Experiment 1 cannot be explained by the horizontal alignment of the dots. Rather, the effect must have been caused by the separate placement of the dots in the left and right visual fields.

## Experiment 3

The bilateral advantage observed in Experiment 1 for numerosities greater than four seems to reflect an advantage of dividing attention between the left and right hemifields as compared to within the same hemifield, as greater numerosities may require greater attention. However, a stimulus-based (“bottom-up”) explanation remains plausible. In particular, retinal stimulation in the bilateral condition may differ from that in the unilateral condition and that could potentially account for the bilateral advantage observed in Experiment 1. For example, previous research has shown that the classical receptive field (CRF) of a visual neuron is surrounded by the non-classical receptive field (nCRF), where stimuli can modulate the responses to CRF [28]. Even if the bilateral and unilateral displays shared identical stimulation in one quadrant, the unique stimulation from the non-shared quadrant could differentially drive the nCRFs of neurons responding to the shared quadrant. Such a stimulus-driven explanation would indeed be more parsimonious than an attention-based (“top-down”) explanation.

Experiment 3 was designed to rule out stimulus-driven explanations such as the one above. In order to match the retinal stimulation between the bilateral and unilateral conditions, our approach was to present dots in all four quadrants on all trials. Prior to the dots, a spatial cue indicated which two quadrants to select for dots enumeration (and which two quadrants to ignore). This experimental design eliminated stimulus-driven differences between the bilateral and unilateral conditions and tested more directly genuine attentional ability.

### Method

#### Participants

Nineteen new volunteers (16 women), aged between 18 and 38 years (mean = 23.6) took part in the experiment.

#### Stimuli and procedure

**Figure 5:**
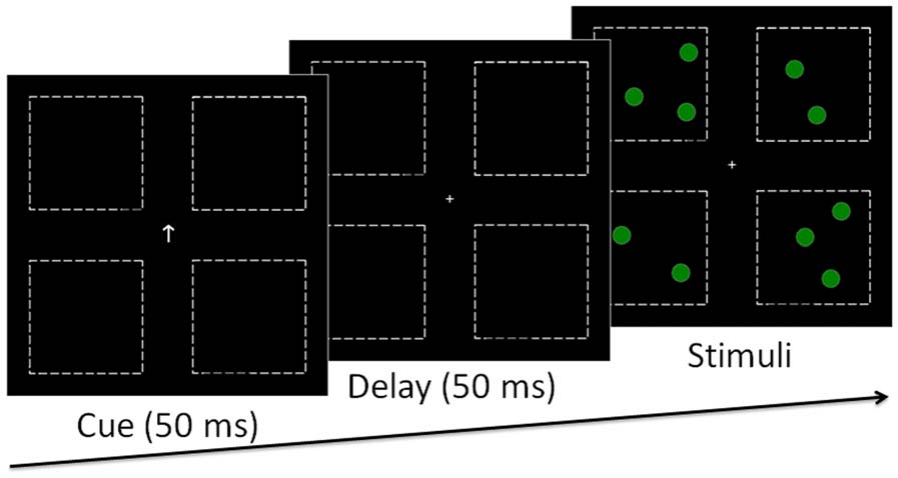
Example of a trial used in Experiment 3. The cue indicates the location of the two quadrants that need to be processed. The example here shows a ‘bilateral’ trial for the upper portion of the visual field.

This experiment replicated Experiment 1 with the following changes: (i) prior to the presentation of the dots, a spatial cue (i.e., a small white arrow of 1.3°×1.3° of visual angle) was centrally presented (50 ms) and indicated which two quadrants to select for dots enumeration; (ii) the cue was followed by a 50 ms blank interval before the presentation of the dots; (iii) dots were then presented in the four quadrants on all trials (see Figure 5). Participants were instructed to enumerate the dots from the two cued quadrants and to ignore those from the two other quadrants. In the unilateral condition, the cue pointed to left or right, whereas in the bilateral condition, the cue pointed to the upper or lower visual field. To equalize retinal stimulations between the two conditions, the number of dots presented in the uncued quadrants always matched the number of dots in the cued quadrants. For example, if three dots were presented in the upper-left quadrant and two dots in the lower-left quadrant, then two dots were presented in the upper-right quadrant and three dots in the lower-right quadrant. Twenty practice trials were followed by 8 blocks of 56 trials (2 conditions ×7 numbers of dots ×32 trials).

### Results and Discussion

**Figure 6:**
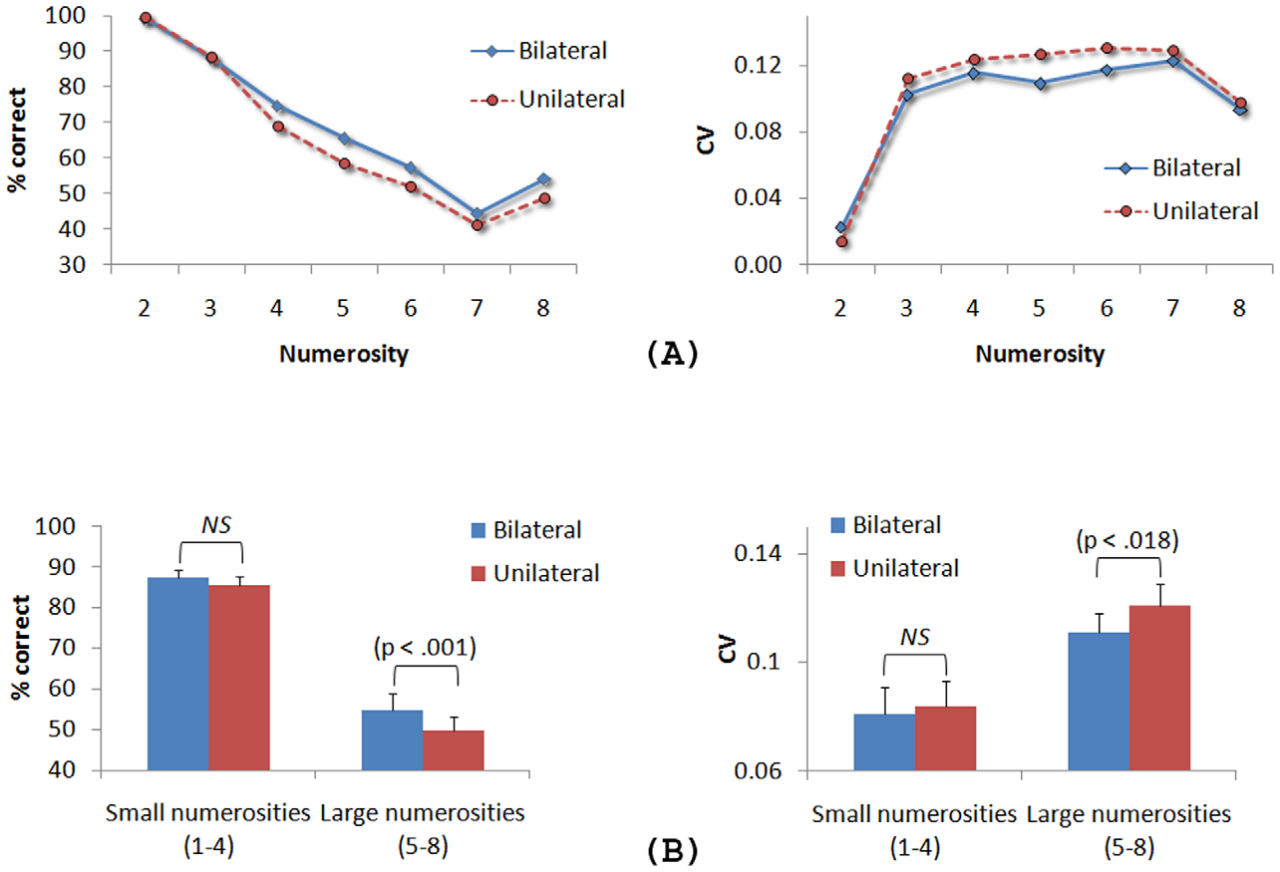
The results of Experiment 3. (A) Percentage of correct responses and coefficients of variation as a function of condition and numerosity. (B) Averaged percentage of correct responses and coefficients of variation divided across small and large numerosities. The error bars represent ±1 standard error of the mean.

A 2 (condition: unilateral, bilateral) ×7 (numerosity: two – eight) ANOVA (repeated measures) on accuracy revealed a main effect of condition, F(1, 18) = 15.42, MSE = 0.006, p<.001, with better performance in the bilateral condition than in the unilateral condition and a main effect of numerosity, F(2.41, 43.32) = 40.41, MSE = 0.098, p<.001, where more errors were made as the number of dots increased. Moreover, despite the lack of significant condition × numerosity interaction (p>.17), the ANOVAs conducted on small (2–4) and large (5–8) numerosities clearly revealed, once again, a condition effect only for the large numerosities, F(1, 18) = 14.61, MSE = 0.007, p<.001, and not for the small numerosities (p>.17) (see Figure 6a).

The 2 (condition) ×7 (numerosity) ANOVA (repeated measures) on CVs revealed no effect of condition (p>.10), a main effect of numerosity, F(3.36, 60.46) = 30.96, MSE = 0.003, p<.001, and no condition × numerosity interaction (p>.49). Figure 6b shows constant CVs of about 0.12 for numerosities greater than three (with the exception of eight) in both conditions (p>.20). Importantly, although the mean CVs for small numerosities (2–4) did not differ between the bilateral and unilateral conditions (p>.69), the mean CVs for larger numerosities (5–8) were significantly smaller in the bilateral condition (CV = 0.11) than in the unilateral condition (CV = 0.12), F(1, 18) = 6.80, MSE = 0.001, p<.018. Similarly to Experiment 1, this indicates less variability, thus higher precision, in responses when the to-be-enumerated items were split across the two hemifields.

## General Discussion

The primary aim of the present study was to examine the effect of dividing visual stimuli across the two hemifields on visual enumeration. Enumeration requires the integration of information into a single quantity and since across-hemifield integration has been found to be more efficient [8], [9] and to occur temporally after the completion of within-hemifield integration [6], [7] one might expect enumeration to be more efficient when the items are presented in one hemifield only. Against this, the present study reveals that visual enumeration is actually more accurate and more precise when the visual items are distributed between the left and right visual fields as when they are all presented within a single hemifield. This bilateral field advantage, however, was only observed when four or more items have to be enumerated, thus when the task was sufficiently attentionally demanding. The study also shows that neither the horizontal alignment of the dots per se (Experiment 2) nor the retinal stimulation differences between the unilateral and bilateral displays (Experiment 3) can account for the observed bilateral field advantage. Rather, this finding is consistent with the notion of independent attentional resources in the left and right hemifields [10], [11] and with recent data that have shown that parallel processing by the neural responses to the left and right hemifields allows more information to be processed [12]–[15].

There are several possibilities how the existence of independent resources in the two hemifields can facilitate the enumeration process. One possibility is that when the number of to-be-enumerated items exceeds the subitizing range, one lateralized quantity is enumerated first while the other is held in short-term memory before being processed for subsequent enumeration and integration with the first value. Recent findings have provided evidence for independent short-term memory representations in the two hemifields [13], which would be necessary for this account.

Another possibility is the existence of two independent enumeration processes, or two pools of attentional resources, one in each hemifield and working in parallel. According to this proposal, the quantities from each visual field are processed independently and simultaneously, predominantly in the contralateral hemisphere. The two resulting quantities are then integrated together via the corpus callosum to provide the final response. Although such a process ultimately requires two quantities to be combined and added together, these costs could be minimal compared to the gain of splitting a large number into two smaller ones in each hemifield. Previous data have indeed suggested that magnitude information is represented in both hemispheres [29]–[32] and that numerical information can be rapidly transferred from one hemisphere to the other during a number comparison task [33], [34]. If the transfer of numerical information across the corpus callosum is fast, the integration of two quantities from different hemifields may also be rapid and efficient. Furthermore, this account would be also consistent with the extensive research by Banich and colleagues [35]–[38] that shows that dividing processing across the hemifields is beneficial when the task is demanding because the subcomponents of the task can be divided between the hemifields and processed in parallel. This account fits well with the present findings, where the bilateral field advantage was observed only when more than four items had to be enumerated, thus when the task was rather demanding in terms of attentional resources. With numerosities less than four, however, there might be sufficient resources to subitize efficiently within a single hemifield removing any split-hemifield differences.

Further research is needed to test those non-exhaustive possibilities. However, whichever account is proposed, the present data strongly demonstrate that the bilateral presentation of information in the visual field can benefit a high-level task that requires inter-hemispheric integration such as visual enumeration.
